# Patient Experience and Acceptability of Six-Lead KardiaMobile ECG Monitoring in Clozapine Clinics

**DOI:** 10.1192/bjo.2026.11472

**Published:** 2026-06-30

**Authors:** Madelaine Bridges, Joanne Rodda, Evelyn Eggleston, Sukhi Shergill

**Affiliations:** Kent and Medway Mental Health Trust, Maidstone, United Kingdom

## Abstract

**Aims:**

This project aimed to explore the experiences and acceptability of individuals prescribed clozapine regarding both traditional 12-lead ECGs and the handheld Kardia Mobile 6-lead ECG device.

**Methods:**

Initial data on 12-lead ECG compliance was analysed across two clozapine clinics using Business Intelligence. 213 patients were prescribed clozapine; however, only 40% (n=85) had an ECG recorded within the previous 12 months.

A quantitative survey using Likert scales was developed and distributed to patients attending clozapine clinics. Participants were offered the handheld Kardia Mobile 6-lead ECG and subsequently completed the patient survey. All participants had previously been offered a standard 12-lead ECG as part of their annual physical health monitoring. A total of 37 patients participated.

Ethical approval was not required as this project was classified as a clinical audit; approval was granted by the KMMH Clinical Audit and Effectiveness Committee (Ref: 6689-25).

**Results:**

Of the 37 participants; 16% reported finding 12-lead ECG’s difficult or very difficult, 38% reported it was neither easy nor difficult, and 46% reported it was easy or very easy. In contrast, 97% of participants reported the Kardia 6-lead ECG to be easy or very easy, with only 3% reporting it as neither easy nor difficult. These findings suggest the handheld ECG was perceived as significantly more convenient and acceptable than the traditional 12-lead ECG.

When asked about preference, 84% of participants strongly preferred the Kardia 6-lead ECG over the traditional 12-lead ECG, and 92% reported they would be more likely to consent to the 6-lead ECG. However, confidence in the accuracy of the Kardia device varied: 21% of participants rated their confidence at moderate or below, while 78% reported high or very high confidence. This indicates that although acceptability and likelihood of consent were high, some uncertainty regarding diagnostic accuracy remains.

**Conclusion:**

The findings demonstrate that the Kardia 6-lead ECG was perceived as more convenient and more acceptable than the 12-lead ECG, with a strong association between ease of use and increased willingness to consent to monitoring. This suggests that the introduction of handheld ECG devices may help address existing barriers to ECG completion in community clozapine clinics and potentially improve annual monitoring rates.

However, it is important to note that the results also highlight while overall confidence in the 6-lead ECG was high, a notable minority expressed uncertainty, emphasising the need for clear patient education and communication regarding the reliability and limitations of handheld ECG devices.

